# Building Better Deep Learning Models Through Dataset Fusion: A Case Study in Skin Cancer Classification with Hyperdatasets

**DOI:** 10.3390/diagnostics15030352

**Published:** 2025-02-03

**Authors:** Panagiotis Georgiadis, Emmanouil V. Gkouvrikos, Eleni Vrochidou, Theofanis Kalampokas, George A. Papakostas

**Affiliations:** MLV Research Group, Department of Informatics, Democritus University of Thrace, 65404 Kavala, Greece; pagoorg@cs.ihu.gr (P.G.); emgkouv@teiemt.gr (E.V.G.); evrochid@cs.duth.gr (E.V.); tkalampo@cs.duth.gr (T.K.)

**Keywords:** hyperdataset, meta-dataset, combining datasets, deep learning, artificial intelligence, skin cancer classification, image dataset fusion, CNNs, visual transformer

## Abstract

**Background/Objectives:** This work brings to light the importance of forming large training datasets with diverse images generated and proposes an image dataset merging application, namely, the Data Merger App, to streamline the management and synthesis of large-scale datasets. The Data Merger can recognize common classes across various datasets and provides tools to combine and organize them in a well-structured and easily accessible way. **Methods:** A case study is then presented, leveraging four different Convolutional Neural Network (CNN) models, VGG16, ResNet50, MobileNetV3-small, and DenseNet-161, and a Visual Transformer (ViT), to benchmark their performance to classify skin cancer images, when trained on single datasets and on enhanced hyperdatasets generated by the Data Merger App. **Results:** Extended experimental results indicated that enhanced hyperdatasets are efficient and able to improve the accuracies of classification models, whether the models are trained from scratch or by using Transfer Learning. Moreover, the ViT model was reported for higher classification accuracies compared to CNNs on datasets with a limited number of classes, reporting 91.87% accuracy for 9 classes, as well as in the case of enhanced hyperdatasets with multiple numbers of classes, reporting accuracy of 58% for 32 classes. **Conclusions:** In essence, this work demonstrates the great significance of data combination, as well as the utility value of the developed prototype web application as a critical tool for researchers and data scientists, enabling them to easily handle complex datasets, combine datasets into larger diverse versions, to further enhance the generalization ability of models and improve the quality and impact of their work.

## 1. Introduction

Deep learning models are known to improve their performance when trained on an enhanced amount of diverse data [1]. Yet, such data are not always easy to collect, especially in the case of sensitive medical data that are subjected to privacy and ethical issues. Moreover, few-shot classification recently introduced the trend of learning new classes from a few samples, aiming for more robust classification models that could not require large training datasets of multiple samples per class. The latter is referred to as the meta-problem, i.e., increasing the complexity of the problem by exploiting the training towards “learning to learn new classes from few examples” [2]. However, datasets to evaluate the meta-problem are scarce. Examples of such datasets are the Omniglot and N-Omniglot datasets [3], which contain 1623 different classes of unique handwritten characters from 50 different alphabets, and the mini-ImageNet dataset, which consists of 100 classes of a total of 60,000 images, all used in few-shot classification tasks. The possibility of merging different smaller datasets to create a larger dataset could serve as a feasible solution to gather multiple and diverse data to train robust few-shot classification models [2].

In practice, similar datasets of the same modality may include different, same, or overlapping classes, which need to be added and sorted when datasets are merged. The latter might be time-consuming in the case of complex datasets that include many classes. Therefore, in this work, a novel image dataset merging application, namely, the Data Merger App, is first presented, capable of merging image datasets automatically, creating so-called HyperDatasets of multiple and diverse class samples, providing a useful tool to researchers interested in the meta-problem. The main scope of this work, however, is to highlight the need for combined datasets, and to investigate the performance of models on such combinations. To this end, in this work, we study the impact of combined datasets on transfer learning and training from scratch, by presenting results through a set of sequential and scalar tests, aiming to investigate the performance of known classification models of various dimensions in the classification problem of skin cancer images when trained on different and diverse enhanced hyperdatasets.

The contributions of this work can be summarized in the following points:

1. Highlighting the need for combined datasets by thorough examination of all possible case combinations regarding the number of classes, and data distributions.
2. Development of a public online application, named the Data Merger App, aiming to easily combine image datasets for classification tasks. The proposed application can automatically merge common classes and add novel classes of the merged datasets, resulting in diverse hyperdatasets of an enhanced number of diverse image classes towards training robust classification models.
3. Investigation of the performance of classification models by using hyperdatasets, i.e., generation and evaluation of realistic class imbalanced datasets for classification studies:
  - Presentation of a specific case study of merging skin cancer image datasets. Evaluation of the developed hyperdatasets by training four Convolutional Neural Network (CNN) models and a Visual Transformer.
  - Investigation of the impact of Transfer Learning and training from scratch by using hyperdatasets of gradually increasing complexity.

A similar approach has been investigated by Triantafillou et al. [2], who proposed a meta-dataset, referring to a hyperdataset, for training and evaluating models. In contrast, in the current work, we do not propose a large-scale dataset consisting of diverse datasets, but we experimentally establish a proof-of-concept regarding merging image datasets towards creating diverse multi-class training hyperdatasets, i.e., meta-datasets, which are essential for training and improving generalization capabilities of classification models. Hopefully, this work aims to demonstrate the benefits of combining multiple datasets, while the proposed application may serve as an essential tool for researchers interested in training and testing their models with data of diverse distributions, multiple classes, and realistic class imbalances.

In what follows, we gradually merge 13 well-known image datasets for skin cancer classification by using the Data Merger App, and we create 13 hyperdatasets of different numbers of images. These hyperdatasets are used to train CNN classification models for the recognition of skin cancer. Advanced neural networks are widely used in medical image analysis [4], providing efficient computer-aided diagnostic tools for classification tasks in multiple areas such as for the brain [5], breast [6], and skin-related [7] detection of medical conditions. Specifically, skin cancer is based on the recognition of symmetries in images; therefore, networks that can efficiently learn specific features on images across multiple orientations are required. Since one of the main capabilities of CNNs is that they are translation invariant, they are considered ideal for learning the discriminative characteristics of skin cancer image data [8].

Therefore, in this work, we additionally explore the performance of four CNN models for skin cancer classification, VGG16, ResNet50, MobileNetV3-small, and DenseNet-161, and a Visual Transformer, by conducting four subsequent tests:

1. First, for training, we use the 13 datasets one by one.
2. Second, we gradually merge the datasets, and we train the same models on the generated hyperdatasets. Transfer learning is employed in both tests.
3. Then, a third test is conducted to study the performance of models when trained from scratch compared to transfer learning, by using a small dataset of a fixed number of classes and a hyperdataset of the same number of classes.
4. Finally, in a fourth test, a Vision Transformer is employed and compared with CNNs for the problem under study, for simple datasets with a finite number of classes and for enhanced multi-class hyperdatasets.

According to this context, the rest of the paper is structured as follows. Section 2 summarizes materials and methods. Section 3 presents the case study of skin cancer classification with the four conducted tests, as explained above. Section 4 provides discussions and future works, while Section 5 concludes the paper.

## 2. Materials and Methods

### 2.1. Merging Datasets

**Figure 1:**
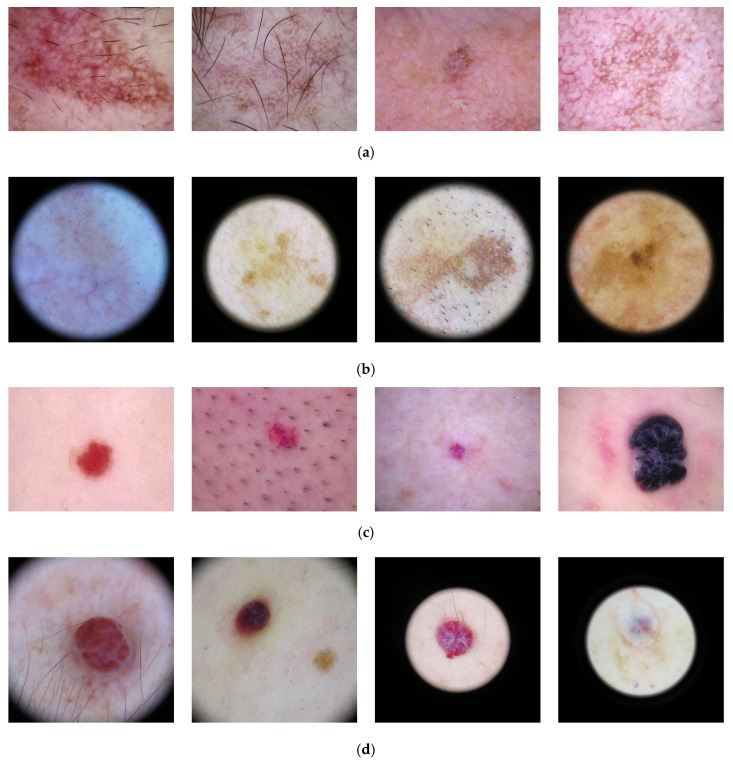
Images from the same classes of different datasets: class actinic keratosis from datasets: (**a**) Skin Cancer ISIC; (**b**) ISIC 2019; and class vascular lesion from datasets: (**c**) Skin Cancer ISIC; (**d**) ISIC 2019.

The combination of multiple datasets to train machine learning models has the possibility of offering several benefits related to the enhanced diversity and richness of the combined data. Given a learning example of N classes c=1, 2, …, N, for a dataset $D_{1}$ of l samples, a classification model learns from the training dataset of k samples, $Tr=\left\{\left(x_{1},\;y_{1}\right),\left(x_{2},\;y_{2}\right),\;\ldots ,\;\left(x_{k},\;y_{k}\right),\;\right\}$ and it is evaluated on a testing set $Te=\left\{\left(x_{k+1},\;y_{k+1}\right),\left(x_{k+2},\;y_{k+2}\right),\;\ldots ,\;\left(x_{l},\;y_{l}\right),\;\right\}$ of (l−k) samples. Consider a different dataset $D_{2}$ with a different number of samples and classes. Moreover, consider that the two datasets contain different images of the same subject, in terms of different capturing conditions such as varying illuminations, changing perspectives, different backgrounds, resolution and focus, or altered images subjected to preprocessing, e.g., filtering, to highlight certain textures or frequency components, etc. Figure 1 includes images of the same classes from two different datasets. Note that the same classes are depicted following a different capturing procedure, resulting in images that may be heterogeneous, i.e., considering the black background being present in the images of one of the datasets.

By combining datasets, the following benefits are theoretically anticipated, while this work aims to investigate practically related challenges, and whether these benefits actually apply.

**Case 1: Datasets with the same number of classes.** In the case of combining two datasets $D_{1}$ and $D_{2}$ of the same m classes, an obvious benefit is related to the increase in the samples (S) per class:

(1) $$S_{D_{1+2}}=\sum_{i=1}^{m}(S_{i_{D_{1}}}+S_{i_{D_{2}})}$$

The resulting benefit is related to dealing with the shortage of data for training DL models with large architectures. In some cases, data are difficult or scarce to collect, due to the rarity of the events, the related high costs, privacy issues, etc. Insufficient data reduce the ability of models to generalize well to new data, resulting in poor performance and overfitting issues. Moreover, small datasets may not provide the true distribution of the data, leading to biased models, and may lack the diversity necessary to capture all features’ variations present in real-world data. Regarding the impact of the meta-dataset on the performance and generalization ability of classification models, the latter is an empirical question related to the distributions of the combined data, as explained in the following subcases.

- **Case 1.1: Similar distributions.** Similar distributions refer to image datasets with slightly different characteristics that are close in their descriptive statistical properties. Combining such datasets can increase training data and provide enhanced learning of the underlying patterns, reduce overfitting by providing diverse examples, balance the class distributions so as to ensure the model is not biased towards a certain class, and smooth out outliers and anomalies leading to robust models of improved performance.
- **Case 1.2: Heterogenous distributions.** While it might be expected that combining datasets and training on more data per class could improve the generalization ability of models, the latter is not certain for datasets with heterogeneous data distributions. In order to take advantage of such data, calibration on the datasets would be necessary so as to bridge large contrasts and produce datasets of similar distributions that could be more efficiently combined. Yet, the challenge is to train models able to adapt to data captured under variable conditions. Heterogenous distributions by depicting the same object might occur due to various reasons, such as variable conditions of data acquisition, lighting variations, different perspectives, resolutions, environmental variations, different backgrounds, camera settings, etc. The combined distribution, which incorporates characteristics of the original datasets, is richer and more diverse, providing the models with a broader set of patterns and varied examples. By combining such datasets, the diversity of the training samples is increased, and the model might learn to generalize across varied conditions. In that case, more data samples can help approximate the true underlying distribution of the data, while they also include rare and edge cases, referring to real-world scenarios. The latter could help the model to generalize better to new data, improving the robustness of the model to capture complex patterns. In this work, we aim to examine the generalization ability of models on combined such data by merging datasets, and by comparing the performance of models on different datasets combinations.

**Case 2: Datasets with different number of classes.** In practice, datasets on the same subject with different numbers of classes are very common, due to different research objectives, different details in annotation, evolution in knowledge leading to newly discovered classes, etc. Therefore, data that someone could not collect from one dataset can be found in another, and combined; this variability is essential towards addressing multiple and diverse research issues that better simulate reality. Combined datasets of multiple and diverse classes can provide realistic class imbalances that are essential for classification-related challenges.

- **Case 2.1: Similar distributions.** As in Case 1.1, the data volume is increased, improving the ability of the model to learn diverse examples from more data from different classes. Similar distributions ensure that the features from the combined datasets align well, leading to more effective features.
- **Case 2.2: Heterogenous distributions.** As in Case 2.1, the combined dataset includes a wide variety of data, with enriched feature distributions, allowing the model to learn more complex patterns. Exposing the model to increased classes of heterogeneous data refers to a challenge of increased difficulty, related to varying class distributions, complex data integration, class imbalances, and biased learning. Such datasets are needed in order to develop new strategies, advanced methodologies, and even robust models able to align features from heterogeneous distributions and ensure effective model training.

**Figure 2:**
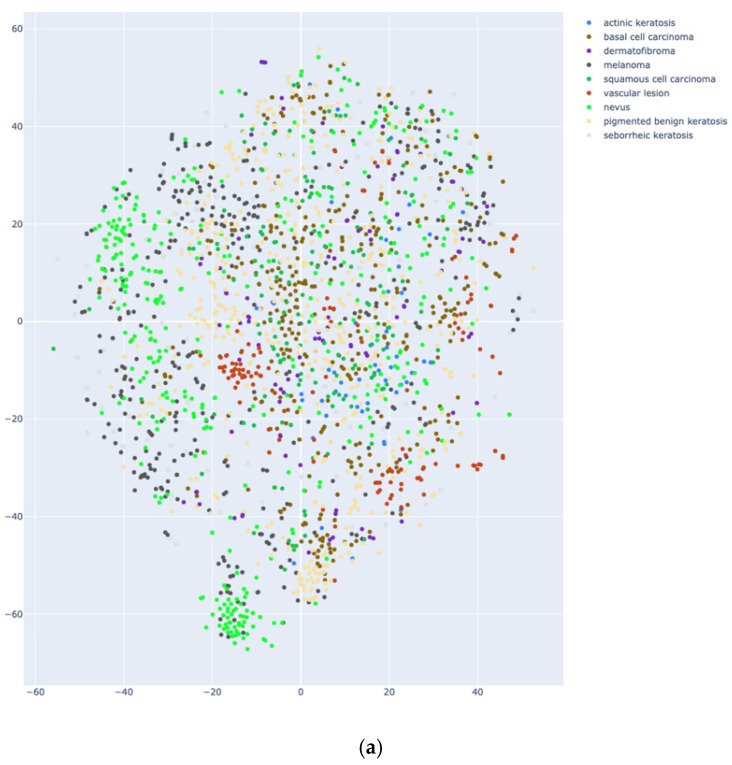

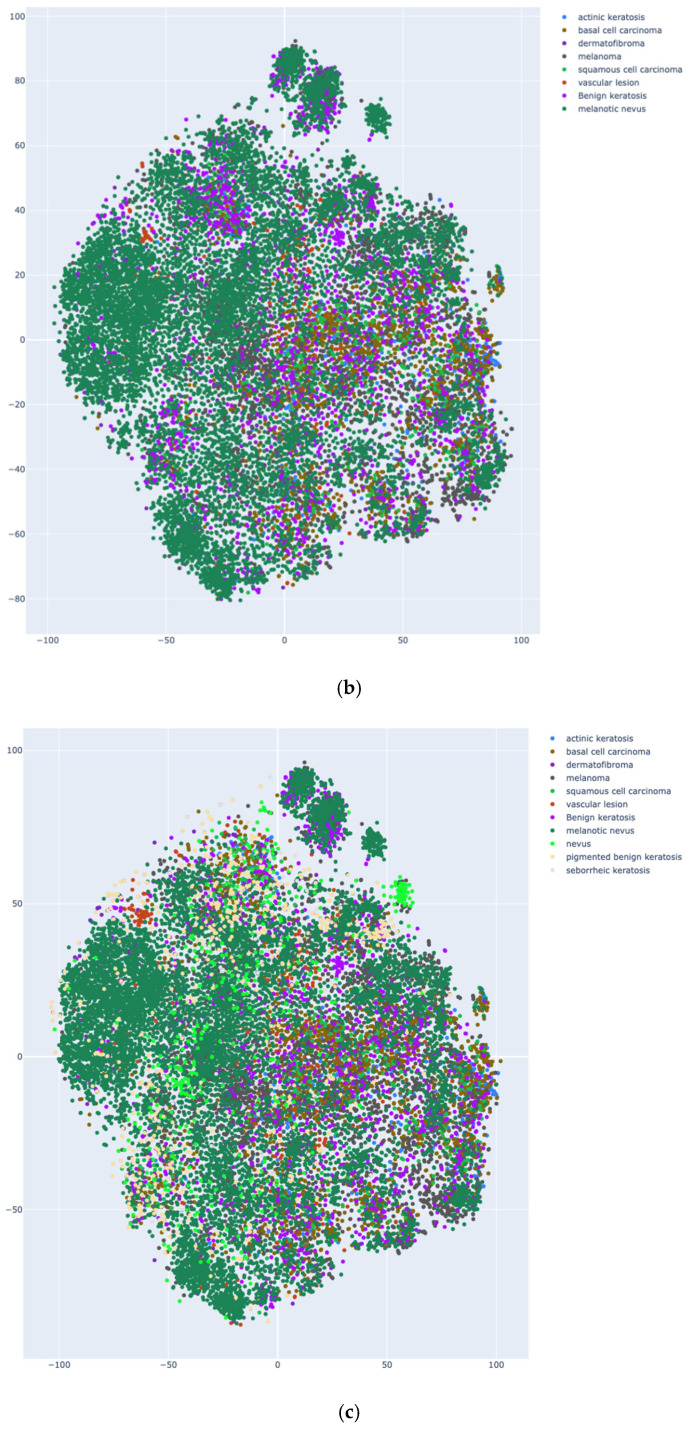
Data embeddings projection method t-SNE with DenseNet161: (**a**) for Skin Cancer ISIC; (**b**) ISIC 2019; (**c**) the combined hyperdataset.

In the following, the intricate relationship between two original datasets and the combined dataset is provided as an illustrative example of a visual representation of data embeddings, shown in Figure 2. Data embeddings are used here to indicate the diversity of the resulting combined hyperdataset. Figure 2a depicts eight classes of Skin Cancer ISIC, and Figure 2b shows nine classes of ISCI 2019 datasets. Note that the two datasets share six common classes. In Figure 1, images of two of their common classes are shown. Figure 2c depicts the embeddings of the combined dataset, including a total of 11 classes, resulting from the combination of the two original datasets. Here, the projection method of t-distributed Stochastic Neighbor Embedding (t-SNE) is selected, to project in 2D space the high dimensional embeddings. Embeddings are calculated with DenseNet161 pre-trained on ImageNet, based on the Explore Data with Embeddings App [9].

The t-SNE projection method indicates that the combined dataset distributions of all classes are mixed; however, common classes appear more concentrated compared to the distributions of the original datasets. Moreover, the increase in the number of different classes reduces the intraclass distance between the classes. Therefore, combined hyperdatasets are expected to increase the raw quantity of data and add meaningful diversity taken from both the original datasets; thus, it is expected that hyperdatasets would be able to challenge the development of more robust classification models to test their performance. The latter may not be feasible in the case of generated synthetic datasets due to the lack of realistic diversity and class imbalances.

### 2.2. Data Merger Application

The Data Merger App (the application will be available for free in GitHub upon acceptance of the manuscript https://github.com/MachineLearningVisionRG/DataMergerApp, accessed on 31 January 2025) was developed to streamline the management and synthesis of large datasets, initially focusing, as a case study, on skin cancer images. However, its versatile functionality extends beyond this specific domain, making it applicable to any dataset containing diverse classes, due to its capability to recognize common classes across all datasets, making it versatile for various applications. The primary motivation for creating this application was to facilitate the creation of hyperdatasets, i.e., comprehensive repositories that consolidate and organize data for various research purposes. This approach enhances data management by providing tools to efficiently merge and organize large datasets, ensuring data are well-structured and accessible. It also promotes collaboration by allowing researchers from different fields to easily share and combine data, fostering interdisciplinary research and collaborative efforts. Additionally, with well-organized and comprehensive datasets, researchers can develop and refine advanced algorithms, leading to significant insights and breakthroughs across various fields of study.

The Data Merger App offers a user-centric interface designed to simplify the management and integration of large volumes of image data. Researchers can select multiple folders and subfolders containing image datasets through built-in file dialogue prompts, facilitating efficient organization and preparation for analysis. The application is meticulously crafted to meet the needs of researchers across various domains, including specialized fields like skin cancer dataset analysis. It ensures accessibility and usability for users of all levels, from seasoned technical experts to newcomers in data management tools. The interface provides intuitive navigation and comprehensive guidance throughout the merging process. By utilizing dynamic widget management within a scrollable canvas, the application efficiently handles large datasets while maintaining optimal performance. This thoughtful design allows researchers to focus on their analysis without being hindered by data management challenges, ultimately enhancing productivity and enabling more impactful research outcomes.

**Figure 3:**
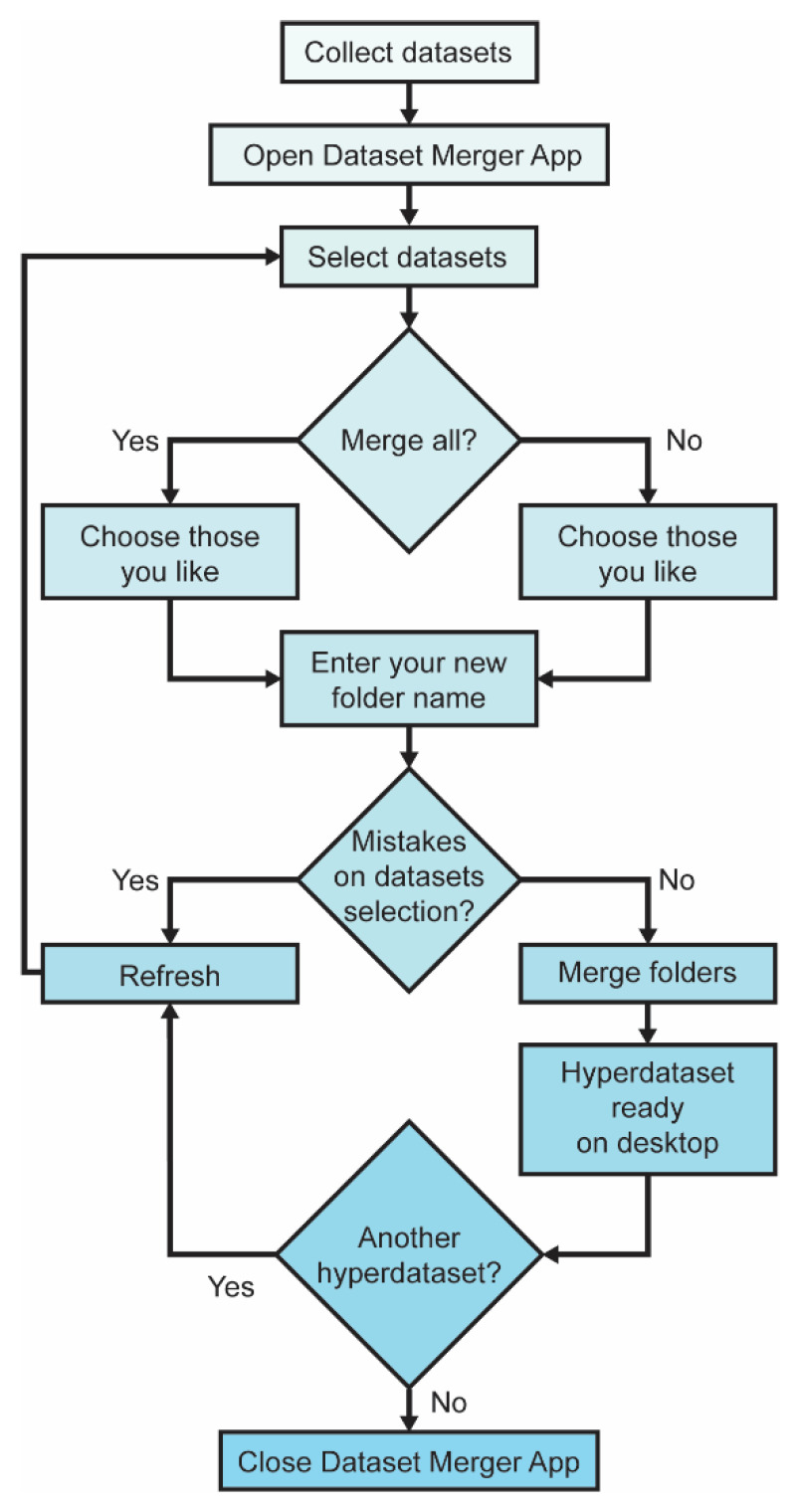
Application usage flowchart.

The Data Merger App is built using Python’s Tkinter framework and integrates backend logic with a responsive frontend interface. Leveraging Python’s versatile libraries such as *os* and *shutil*, it efficiently manages file operations and directory structures, crucial for organizing and merging diverse datasets. Figure 3 illustrates the Data Merger App usage flowchart.

## 3. Case Study

Our case study focuses on the classification of skin cancer. Skin cancer is one of the most important cancer types with an increasing incidence in recent decades. In Europe, melanoma skin cancer ranks as the sixth most commonly occurring cancer type and it is the 17th in global prevalence with cancer-related mortality [10] in Europe, making its accurate and timely diagnosis vital.

The skin cancer datasets are synthesized from dermoscopic images. Many datasets are provided by the research community. Researchers have applied strategies based on splitting, merging, clustering, and classification to identify and treat skin cancer [7,11].

The International Skin Imaging Collaboration (ISIC) dataset formed the basis for the first public large-scale benchmark dataset of dermoscopic images in 2016 [12,13], currently counting more than 20,000 images, widely used for skin lesions analysis and detection of melanomas. PH2 is also a well-known public archive of dermoscopic images of melanoma [14]; yet PH2 is very small, including only 200 images. On the one hand, a deep learning model trained on a small number of samples may not perform well during testing. Limited training data often suffer from class imbalance, and may lead to overfitting, a lack of generalization, and difficulties in learning complex patterns such as those of different types of skin cancers. On the other hand, if the training datasets are too large, there are concerns about how well and efficiently a model can learn [15]. Large training samples are harder to handle, require significant computational resources, and can result in longer training times and higher related costs. Moreover, imbalances in classes can be more intense, with some classes having significantly more samples compared to others, leading to biases towards the majority classes and subsequently to poor model performance on the minority classes.

Therefore, the scope of this case study is to observe and record the performance of models on extended datasets, towards concluding whether the combination of multiple datasets by using the Data Merger App would be, after all, a good practice for optimizing models’ performance.

Therefore, in what follows, well-known benchmark datasets of skin cancer images are collected and used to train four different Convolutional Neural Network (CNN) classification model architectures. Then, with the proposed Data Merger App the collected datasets are merged into larger datasets. The training process is repeated, trying to evaluate the behavior of the models on the new large datasets.

The case study is concluded by examining the state of maximizing the most commonly occurring skin cancer categories around the collection of datasets, by using the proposed Data Merger App for the merging process.

### 3.1. Used Datasets

For our case study, 13 well-known image datasets of RGB skin cancer images based on the bibliography were selected. The primary objective was to ensure that all datasets were relevant to skin cancer and shared common compositional elements, as well as they were free (open) and easy to obtain. Given that skin cancer is a complex issue, these datasets can potentially be enriched with additional elements beyond their initial representation, such as clinical metadata, histopathological annotations from biopsies, and more. However, for the purposes of our research, we chose to focus solely on skin cancer types and their representation.

Table 1 summarizes key information about the 13 selected datasets, highlighting their significance and contribution to the field. Download links in Kaggle and/or referenced works are also provided in the table. The datasets vary in the number of classes and samples that they contain; however, there are overlapping classes across them. This overlap is particularly advantageous for the merging process of these datasets towards creating their augmented versions. Each class corresponds to a specific category of skin cancer; thus, by uniting these datasets, the overall dataset’s robustness and diversity can be enhanced, potentially improving the effectiveness of AI-driven skin cancer classification systems, as examined in the following.

First, the datasets are prepared for the training process. All images of all datasets were resized to a resolution of 224 × 224 pixels to be consistent and compatible with the selected DL architectures, and normalized to a range between [0, 1] to stabilize training, leading to faster convergence and reducing bias. No augmentation was applied to any of the initial datasets of Table 1. The latter was intended so as to maintain their original size. This approach aimed at allowing us to accurately assess whether merging the datasets would yield positive outcomes.

### 3.2. Classification Models

In what follows, we explore the potential of four CNN models, VGG16 [33], ResNet50 [34], MobileNetV3-small [35], and DenseNet-161 [36], for skin cancer classification, by first conducting two tests: (1) by using the 13 datasets one by one, referring to the individual training of the models; and (2) by merging them with the Data Merger App, referring to the merged training. Transfer learning is employed in both tests. Then, a third test is conducted, (3) to study the performance of models when trained from scratch compared to transfer learning, by using a small dataset of a fixed number of classes and an enhanced dataset of the same number of classes formed by using the Data Merger App. Finally, in a fourth test, (4) Vision Transformers are employed and compared with CNNs for the problem under study, for simple datasets with a finite number of classes and for enhanced datasets formed by using the Data Merger App with multiple classes.

Four distinct CNN classification model architectures were selected, each varying in size (number of layers and parameters) and complexity. This scalable model structure allows for thoroughly assessing dataset performance across small, medium, and large models. Additionally, one of the selected models, MobileNetV3-small, is optimized for mobile devices or embedded systems, offering valuable insights into robustness in resource-constrained environments.

All models were trained using the PyTorch library on Google Colab Pro. The training setup included a categorical cross-entropy loss function, gradient descent optimization, a learning rate of 0.001, and a batch size of 32 over 25 epochs. To ensure that the training batches represented a well-distributed sample of the datasets, weighted random sampling was utilized. Finally, the datasets were split with a 70:20:10 ratio, allocating 70% of the samples for training, 20% for validation, and 10% for testing.

### 3.3. Tests 1–4: CNNs

In this work, four tests are conducted, aiming to highlight the importance of merging datasets in progressing the classification task:

- First, we use transfer learning and the 13 datasets for training the models one by one. This approach provides a comprehensive understanding of how each model performs across the different datasets.
- Second, we gradually merge the datasets, and we train the same models on the generated hyperdatasets, using transfer learning. This approach aims to generate new, extended datasets, by gradually combining the datasets to form a larger, unified set, and comparatively evaluate the classification performance of the deep learning models.
- Third, we study the performance of models when trained from scratch compared to transfer learning (previous two tests), by using a small dataset of a fixed number of classes and a hyperdataset of the same number of classes. More specifically, given the results of the previous two tests, the focus of the third test is on training the DL models by using the dataset that contains the most common classes between all selected datasets. This dataset aims to serve as the foundation, upon which the proposed Data Merger App is applied to extend the dataset for the common classes. This experimental approach is designed to keep the number of classes low while increasing the number of samples per class, thereby enabling the study of the models’ ability to effectively learn and differentiate between various types of skin cancer in extended datasets.
- Fourth, a new popular model architecture, a Vision Transformer, is also used for simple datasets with a finite number of classes and enhanced multi-class hyperdatasets, towards studying its performance for the problem under study and comparing it with the performance of CNNs.

#### Test 1: Individual Training

The first phase of the experiments involved training each model on all 13 datasets individually. This approach provides a comprehensive understanding of how each model performs across different datasets. For this phase, transfer learning is employed, leveraging models pre-trained on the ImageNet dataset.

Accordingly, the convolutional layers in all models were frozen, with only the final classification layers left trainable. This strategy allows us to focus on the specific skin cancer classification task while retaining the general visual features learned from the broader ImageNet dataset. The following four tables, Table 2, Table 3, Table 4 and Table 5, summarize the performance metrics of the four models after being trained on the 13 datasets, including training and testing accuracy, F1-score, sensitivity, and specificity.

The completion of model training across all 13 datasets individually has yielded results that, in many cases, are quite similar. DenseNet-161 stands out, achieving the best test accuracies by using the majority of datasets, and an average test accuracy of 77% across all datasets. It is followed by ResNet50 with 74%, and VGG16 and MobileNet both with 72%.

In general, the larger models, such as DenseNet and ResNet, demonstrate superior performance, while the smaller models, like VGG16 and MobileNet, deliver poorer results. MobileNet, in particular, despite being the smallest of the four models, performs satisfactorily, displaying better accuracies for some datasets compared to other models, such as for Skin Cancer ISIC and SKINL2.

In cases of datasets of two classes, such as the Melanoma or Malignant vs. Benign datasets, the models easily achieve high performance, with accuracy rates exceeding 90%. Conversely, when the number of classes increases, accuracy typically drops to between 50% and 70%.

Finally, among the four models, it is worth highlighting that for the Skin Cancer Types Images dataset, VGG16, ResNet50, and DenseNet161 achieved an accuracy of 100%, while MobileNet achieved 97%. The latter can be attributed to the quantity and quality of the dataset, including a balanced number of images of all classes, as well as to the models’ tuning, appearing to be highly adaptable.

### 3.4. Test 2: Merged Training

In the second conducted test, the Data Merger App is utilized to generate new, extended datasets. As previously mentioned, the scope of this test is to combine the datasets to form a larger, unified set, and evaluate the classification performance of four deep learning models.

The Data Merger App efficiently managed the merging process, ensuring that, in instances of common classes, the samples were appropriately aggregated. The merging process was initiated by integrating one dataset at a time, without following any specific order or pattern; the selection of datasets for merging was additive and entirely random. Through this process, 12 new augmented datasets were generated (Merged datasets 1–12).

Then, the training process of all four deep learning models was repeated for all 12 newly created hyperdatasets. The training was performed using the transfer learning technique, with a categorical cross-entropy loss function, gradient descent optimization, a learning rate of 0.001, a batch size of 32, and 25 epochs. Table 6 includes the performance results of the models for all merged dataset cases.

The results included in Table 6 highlight the models’ inability to meet the demands of datasets with a large number of classes. As the number of classes increases, the accuracy of the models decreases. Smaller models are more vulnerable to the increase in classes, while larger models display small performance differences. Overall, it is reasonable to conclude that there is a significant performance gap correlated to model size, as differences in accuracy can reach up to 10%. Our general observation is that CNN models, irrespective of their size, struggle to perform effectively on datasets with a high number of classes. In general, when combining datasets, the distribution of classes tends to become imbalanced, especially as new classes are added. Moreover, the complexity of the classification task is gradually increasing, making it difficult especially for light models to distinguish between a large number of classes. Another reason is due to the inconsistency between different datasets, referring to the variations in image quality and resolution, that might challenge the models.

### 3.5. Test 3: Training from Scratch

In the third experimental step, given the results of the previous two tests, the focus is on training the four models by using the dataset that contains the most common classes between all selected datasets. This dataset will serve as the foundation, upon which we will apply the proposed Data Merger App to extend the dataset, but only for the common classes.

This experimental approach serves to keep the number of classes low while increasing the number of samples per class, thereby enabling the study of the models’ ability to effectively learn and differentiate between various types of skin cancer in extended datasets by using the proposed application.

The process of identifying the dataset with the most common classes is performed by using a Python script designed to compare the datasets and determine the shared classes. In our case, the dataset identified as having the most common classes with all the rest of the selected datasets is the “Skin Cancer ISIC” dataset. More specifically, the dataset appears to have 20 common classes with all 13 datasets.

Up to this point, the training efforts have relied on transfer learning. However, in this phase of the experiments, all four models were trained from scratch, without utilizing transfer learning. More specifically, our models will begin with no prior knowledge and will be trained entirely from scratch on the “Skin Cancer ISIC” dataset and its augmented subsequently formed dataset by using the Data Merger App, namely, the enhanced dataset. This approach aims to assess whether the dataset merging technique would yield positive results when the models are trained from scratch compared to the transfer learning approach. The same configuration for both training methods is maintained, employing a categorical cross-entropy loss function, gradient descent optimization, a learning rate of 0.001, a batch size of 32, and 25 epochs. Results for the Skin Cancer ISIC dataset are included in Table 7, while for the enhanced Skin Cancer ISIC dataset, they are included in Table 8.

Results indicate that the Transfer Learning technique consistently yields better outcomes in both scenarios, for the simple and the enhanced dataset. This is particularly evident in the first training scenario with the simple dataset, where the dataset contains a limited number of samples per class. In these instances, training the models from scratch fails to achieve accuracy rates, hardly approaching 50% with the larger models, ResNet and DenseNet. In contrast, the smaller models, VGG and MobileNet, exhibit accuracy levels below 30%. However, by applying Transfer Learning, the accuracy of all models is improved, reaching up to 66%, for DenseNet.

Classification results are further enhanced when using the augmented version of the dataset. In that case, even models trained from scratch demonstrate significantly improved performance, with an accuracy of more than 50% in most cases. Although there is additional improvement with the use of Transfer Learning, it is not substantial. The latter leads to the conclusion that augmenting the number of samples per class in a dataset has a positive impact on the performance of models, while the Data Merger App proved its feasibility to provide enhanced data for the problem under study. Moreover, Transfer Learning by leveraging pre-trained models that have already learned the features of the problem from large datasets can benefit the classification process and lead to even better results.

**Figure 4:**
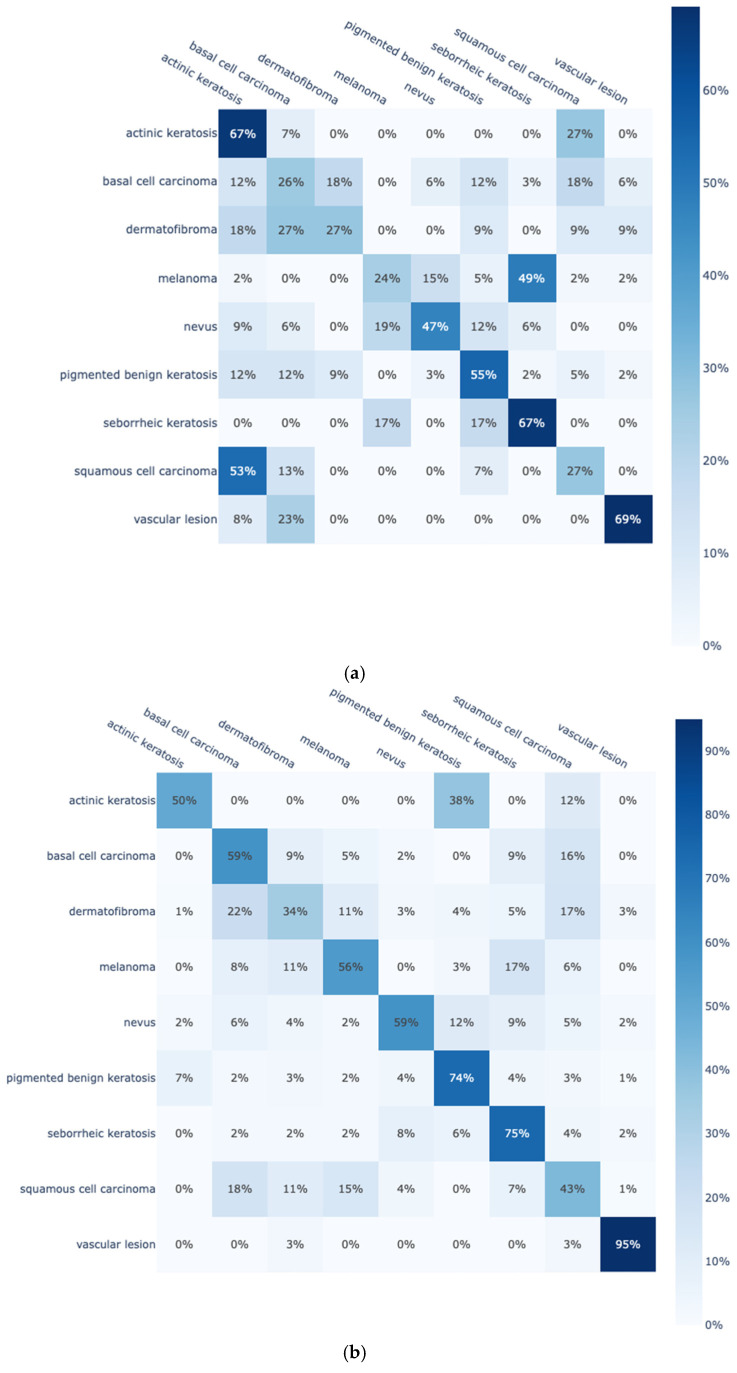
DensNet-161 confusion matrix trained from scratch on (**a**) the Skin Cancer ISIC dataset; and (**b**) the enhanced Skin Cancer ISIC dataset.

**Figure 5:**
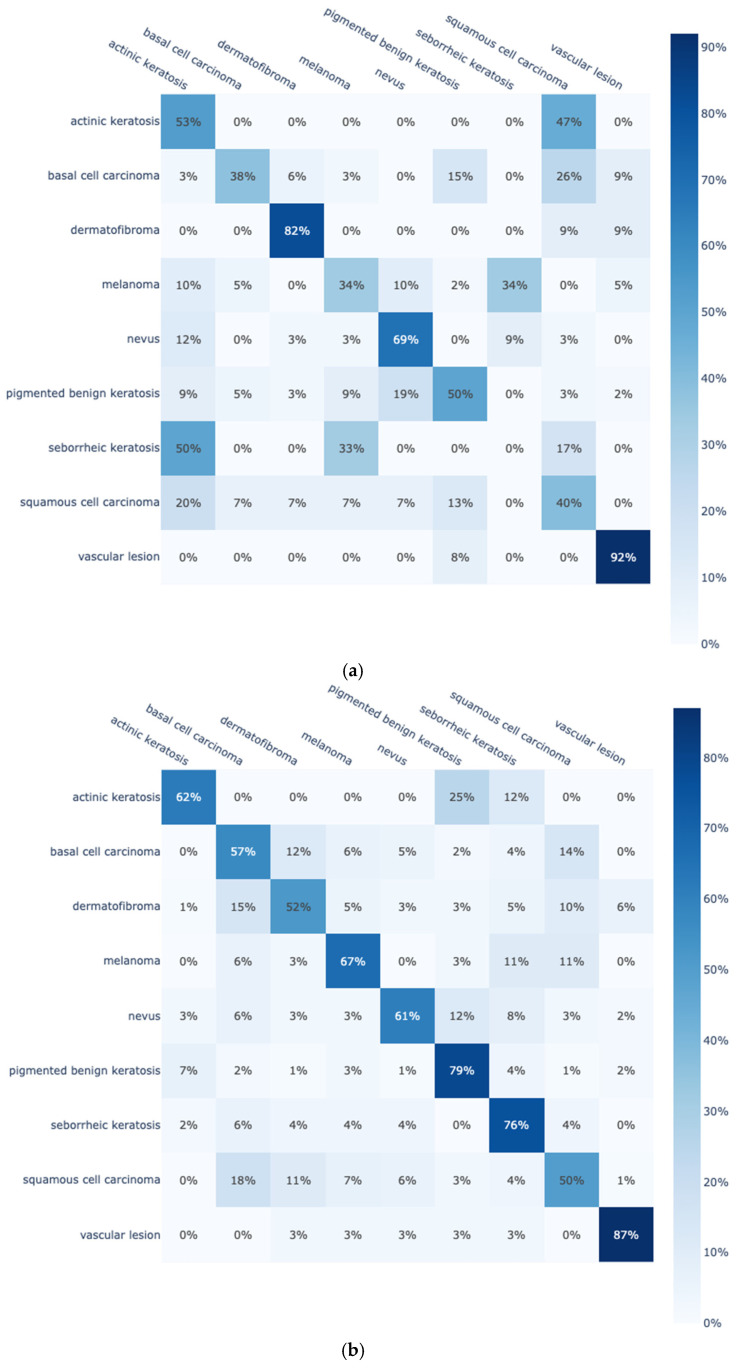
DensNet-161 confusion matrix trained with transfer learning on (**a**) the Skin Cancer ISIC dataset; and (**b**) the enhanced Skin Cancer ISIC dataset.

To further enhance the presentation of findings, two representative confusion matrices are presented in Figure 4 for the best-performing model DenseNet-161 trained from scratch on the original (Figure 4a) and on the enhanced Skin Cancer ISIC dataset (Figure 4b). From the confusion matrices of Figure 4, it is clear that by using the enhanced dataset, DensNet-161 is able to greatly improve the classification rates for eight out of the nine classes. In the remaining class, the classification rate remains relatively stable. Moreover, two confusion matrices are also included in Figure 5 for the best-performing model DenseNet-161 trained with transfer learning on the original (Figure 5a) and on the enhanced Skin Cancer ISIC dataset (Figure 5b). Similar observations are noted; by using the enhanced dataset, classification rates are improved in most of the classes due to the increase in training data per class.

To better translate the confusion matrices, the samples per class for the original and the enhanced Skin Cancer ISIC dataset are included in Table 9. Note that class “pigmented benign keratosis” remains the same in both datasets due to only being existent in the original Skin Cancer ISIC dataset. In general, classes that are greatly enhanced display better classification performances whether the model is trained from scratch or with transfer learning.

**Figure 6:**
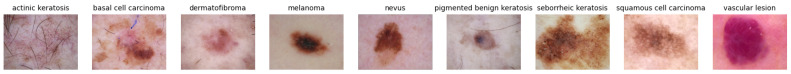
Indicative images for all nine classes of the Skin Cancer ISIC dataset.

Even in the case of transfer learning and with enhanced datasets, the assignment of samples to other classes is observed, as shown in Figure 5b. This is due to the fact that some classes are very similar to each other, which makes the problem particularly difficult. Figure 6 shows images from each of the nine classes of the Skin Cancer ISIC dataset, where a particular similarity between classes can be observed. More specifically, classes “dermatofibroma”, “basal cell carcinoma”, and “squamous cell carcinoma” are visually very similar to each other. The same is also reflected in the results included in all confusion matrices, where samples from these three classes are misattributed between each other. Regarding the best-performing case of the enhanced dataset with transfer learning of Figure 5b, the model achieved a classification rate below 60% only in these three classes: 57% for “basal cell carcinoma” (12% and 14% were misclassified as “dermatofibroma” and “squamous cell carcinoma”, respectively), 52% for “dermatofibroma” (15% and 10% were misclassified as “basal cell carcinoma” and “squamous cell carcinoma”, respectively), and 50% for “squamous cell carcinoma” (18% and 11% were misclassified as “basal cell carcinoma” and “dermatofibroma”, respectively).

### 3.6. Test 4: Vision Transformer

All previous tests have employed CNNs, a well-established solution for image classification tasks. However, the machine learning community is nowadays increasingly focused on a new model architecture known as Transformer [37], which has been instrumental in the development of Large Language Models (LLMs) [38]. The advent of transformers has also significantly impacted the field of computer vision, particularly through Vision Transformers (ViTs) [39]. ViTs leverage the self-attention mechanism [40], which enables the model to monitor different regions of the input data based on their relevance to the specific task. This mechanism enhances model accuracy, allows for efficient handling of variable-length inputs, and improves interpretability and explainability.

Therefore, in the fourth test, a large ViT model, pre-trained on ImageNet-21k and refined on ImageNet 2012, was fine-tuned to be used for the problem under study. The Hugging Face transformer library was employed, utilizing a learning rate of 0.00002, a batch size of 10, and training over 10 epochs. All tests involving the ViT model were incorporated into the fourth and final phase of the training as a supplement to the Transfer Learning practice. Training a VIT model is rendered unfeasible given the available dataset, as such architectures demand an extensive volume of data for effective training. Consequently, fine-tuning the model was the only viable approach.

Therefore, as a follow-up of the previous experiments, the ViT model was trained on the “Skin Cancer ISIC” dataset and its augmented subsequently formed dataset by using the Data Merger App, namely, the enhanced dataset, of test 3. Classification results are included in Table 10.

An analysis of the ViT model results clearly highlights its superiority. Even on training with a limited number of samples, the model achieved an accuracy close to 80%, and when the dataset was enhanced, the accuracy reached nearly 92%. The Data Merger App efficiently provided an enhanced dataset that managed to further boost the model’s performance.

Results of Table 10 referring to ViT can be compared with those of Table 7 and Table 8 referring to the CNNs performance, for the same problem. The best-performing CNN model (DenseNet-161) reported 50% accuracy on the same Skin Cancer ISIC dataset (Table 7), and 61% on its extended version (Table 8).

In general, ViTs have shown impressive performance in classification tasks [41], yet they come with computational trade-offs, including extensive computational resources and training time. It is known that ViTs require a large amount of data to achieve comparable to a CNN’s performance [42], while they have higher computational requirements and may be more time-consuming to train. Thus, they require substantial memory to deal with large input images, while their inference speed could be slower compared to simple CNN models, especially on resource-constrained devices oriented for real-time and real-world applications. Therefore, in this work, their primary limitation was in the extended training time, which, however, at only 10 epochs, could provide accuracies comparable and much higher to that of CNNs. In general, the prolonged training duration of ViTs is attributed to their integrated attention mechanism, which allows the model for global context understanding.

To address these issues in real-world settings, techniques like pruning, quantization, and knowledge distillation can be employed to reduce the size and computational requirements of the model, without affecting its performance [43]. Efficient ViT variants, e.g., with partial attention mechanisms [44], and hybrid models combining ViTs with lightweight CNNs [44], could also help to balance computational costs and accuracy. For enhanced inference speed, edge devices could be used to implement ViTs, while transfer learning can be beneficial, to pre-train the ViT models and fine-tune them on specific tasks, such as demonstrated in this work.

Nevertheless, questions arise regarding the performance of ViTs when applied to datasets with a large number of classes. Thus, to study the behavior of ViTs in a large number of classes, a final training was conducted in our test series, aiming to compare the performance of a ViT model against a CNN model on an extended dataset delivered from the Data Merger App comprising 32 classes, referring to test 2 and Merged dataset 12, of Table 6. The best-performing CNN in that case was DenseNet-161, and it is the one compared with the ViT for the same task. Table 11 summarizes the results.

The results of Table 11 clearly indicate the superiority of the ViT model compared to the best-performing CNN in the case study of the extended dataset of 32 classes. In both cases, the models were trained using the same extended dataset. However, the VIT model achieved an accuracy close to 60%, whereas the CNN model reached only mid-level performance.

Notably, the ViT model achieved this accuracy with only 10 epochs, compared to the CNN model, which required 25 epochs to reach an accuracy of 37%. Additionally, it is important to highlight that the ViT model is trained using a smaller batch size and a significantly reduced learning rate.

While similar parameters could be applied to the CNN model, so as to be directly comparable, the smaller batch size could lead to underfitting, and the reduced learning rate could impact training duration or potentially cause overfitting. In conclusion, despite the longer training times, the ViT model outperformed traditional CNN models in terms of accuracy on both limited datasets, as well as in the case of extended datasets of multiple classes.

## 4. Discussion and Future Improvements

In this work, an image dataset merging application is proposed and evaluated among a set of sequential and scalar tests, aiming to investigate the performance of known models of various dimensions in the classification problem of skin cancer when trained on different and diverse datasets.

Merging image datasets aims to gather information from multiple sources towards creating a more comprehensive and accurate representation of real data, including realistic class imbalances for classification purposes. The scope is not just to create larger datasets, since the latter can be achieved through data augmentation techniques, but to create hyperdatasets of diverse merged datasets of real images of multiple classes, to create more complex and difficult datasets to design and train robust models efficiently.

Augmented images generally maintain the same distribution as their image sources. In contrast, different images display different distributions, due to different lighting conditions, angles and perspectives, backgrounds and environments, and different capturing sensors and settings. All the above create variations that result in different data distributions. The resulting variability is necessary to train and test efficient machine learning classification models that can perform as universal approximators. A diverse dataset can improve the generalization ability of the classifier, making it robust to unseen image data. Therefore, merging datasets with different distributions and numbers of classes results in hyperdatasets with an increased number of different real images per class, and an increased number of classes. Such datasets are needed to design classifiers for a broader range of variations and features and improve their generalization abilities. The latter is crucial in real-world applications, where, in most cases, models are trained on laboratory datasets and need to be retrained later in the field on problem-specific data. Thus, hyperdatasets can be used to pre-train models that can be then fine-tuned on problem-specific tasks, leveraging the feature representations learned from the multiple real diverse data of hyperdatasets.

Class imbalances do not need to be handled, as the proposed hyperdataset creation application serves to provide datasets for challenging classification problems. In case, however, class imbalances need to be handled, so as to ensure all classes of a hyperdataset are equally well-presented, to avoid biases, and to conclude better models’ performances, common augmentation techniques can be applied in a subsequent step so as to balance the classes of the hyperdataset.

For this reason, as for future work, many new features are planned for deployment in order to significantly improve the Data Merger’s App capabilities and give researchers more potent tools for preprocessing and data augmentation. The goal of these enhancements is to make datasets more flexible and robust through the use of image augmentation, which is especially useful for creating and evaluating machine learning models. The ability to rotate photos by certain degrees is one of the primary anticipated features. By doing this, the dataset will be more diversified, and the models will be able to adapt to various orientations of the same object. Note that in radiology, images of abnormalities appear at different orientations, therefore applying rotations to the training image dataset can help the model become rotation invariant, i.e., to be able to better identify abnormalities regardless of their position in the image. Furthermore, by mirroring images, horizontal and vertical convolutions will be created, thus doubling the dataset and offering more diverse information for model training. In the same direction, scaling capabilities can be included so that researchers can change the size of photos. For the purpose of building datasets that imitate various distances or resolutions, the latter capability is essential, ensuring that the models can recognize lesions at different sizes and scales, leading to more accurate detections. Future work on the Data Merger App also plans to allow users to crop photographs to highlight particular regions of interest, which is helpful for emphasizing significant details in an image. Another important component will be adjusting the brightness and contrast of the photos. By simulating various illumination conditions, the models will become more resilient to real-world situations. Similarly, altering the color of the photos will contribute to the creation of varied data collection that replicates different lighting situations and camera settings. In order to create datasets with noisy images and allow training models to be resilient to such circumstances, Gaussian noise can be added. Such types of common noise can simulate real-world imperfections caused by sensor noise, lighting variations, and captured artifacts, and can help the model learn to recognize lesions even in images that are imperfect or degraded. Lastly, adding blur to photos can assist in creating models that can handle less-than-ideal image quality by simulating different degrees of out-of-focus situations.

To this end, the Data Merger program aims to optimize data management and make it possible to create more comprehensive and diverse datasets, enhancing its capabilities by integrating additional features in the future as presented above. Note that, apart from its specific application in this work in skin cancer research, the Data Merger App can offer broad utility across various research topics and disciplines. Its adaptable architecture and customizable features make it suitable for any researcher seeking to organize, merge, and prepare datasets for analysis in fields ranging from medical imaging to environmental science and beyond.

The ability to easily create hyperdatasets aims to result in the development of more potent and precise models across a range of study domains. Experimental results indicate benefits when models are trained on gradually larger combined datasets, regardless of the number of classes, which, however, tends to disappear when the number of datasets is excessively increased, as in the case of training on all datasets. It is observed that multiple classes increase the complexity of the problem and make training difficult since increased difficulty leads to decreased performance. Training from scratch is beneficial in some cases, however, transfer learning leads to better model performance in most cases, especially for combined datasets with a multiple number of classes. Results of the conducted experiments indicate that more research needs to be focused on methods to exploit combined data that might be heterogeneous towards better generalization of models to unseen classes. The conducted experiments reveal that learning across gradually combined training datasets does not necessarily result in the theoretical and desired benefits in most cases, especially when datasets contain different classes. The examined models do not always improve when trained on hyperdatasets. However, such combined datasets obtain realistic class ratios and are useful to challenge the development of more robust models, setting an important research goal for future research in the field.

## 5. Conclusions

In this work, an online application to create large-scale, diverse, and realistic datasets, e.g., hypermarkets, for classification purposes is presented, namely, the Data Merger App. To rigorously evaluate the efficacy of the proposed application, hyperdatasets generated through dataset merging were employed to train advanced deep learning models, including four Convolutional Neural Networks (CNNs) and a Vision Transformer (ViT). This comprehensive evaluation aimed to assess multiple dimensions: the influence of dataset merging on model performance, the challenges and opportunities associated with increasing the number of classes in classification tasks, and the comparative effectiveness of transfer learning vs. training models from scratch. The experimental setup highlights the interplay between diverse data synthesis and state-of-the-art architectures in improving generalization and classification accuracy. The results indicated that hyperdatasets do not necessarily improve the performance of models, and multiple classes increase the complexities and challenge the training, while transfer learning may lead to better accuracies especially across datasets with multiple classes. ViT overall displayed the best performance, reaching an accuracy of 91.87% for 9 classes and 58% for 32 classes. Future work suggests improvements to the Data Merger App so as to include additional augmentation capabilities, as well as further investigating the classification capabilities of models pre-trained on hyperdatasets.

## Figures and Tables

**Table 1:** The selected datasets are ordered chronologically.

No.	Ref.	Name	Year	No. of Classes	No. of Images
1	[16]	Skin Cancer ISIC	2019	9	2357
2	[17]	Skin Lesion Images for Melanoma Classification (ISIC 2019)	2020	8	25,331
3	[18]	SKINL2	2019	8	250
4	[19,20]	HAM10000	2018	7	10,015
5	[21,22]	Mpox Skin Lesion Dataset Version 2.0 (MSLD v2.0)	2023	6	755
6	[23]	Skin Cancer Types Images	2022	4	400
7	[14,24]	PH2	2013	3	200
8	[12,25]	Melanoma Detection (ISIC 2017)	2018	3	2750
9	[26,27]	Dermatology database used in MED-NODE	2015	2	170
10	[28]	Melanoma	2018	2	10,015
11	[29]	Malignant vs. Benign	2019	2	1800
12	[30,31]	Monkeypox Skin Lesion Dataset (MSLD)	2022	2	228
13	[32]	ISIC 2019 and 2020 malignant or benign	2022	2	11,400

**Table 2:** Classification results using VGG16 with individual dataset training. Datasets are sorted by the number of classes.

Dataset Name	No. of Classes	Training Accuracy	Testing Accuracy	F1-Score	Sensitivity	Specificity
Skin Cancer ISIC	9	51%	54%	0.4685	0.5307	0.4637
ISIC 2019	9	49%	48%	0.3370	0.3414	0.5003
SKINL2	8	64%	67%	0.5363	0.5593	0.6074
HAM10000	7	71%	73%	0.4094	0.4859	0.3722
MSLD v2.0	6	78%	69%	0.6360	0.6330	0.7186
Skin Cancer Types Images	4	92%	100%	1.0000	1.0000	1.0000
PH2	3	68%	50%	0.4754	0.5259	0.5166
ISIC 2017	3	62%	62%	0.5693	0.5565	0.6267
Dermatology database used in MED-NODE	2	81%	66%	0.5846	0.6071	0.5833
Melanoma	2	91%	92%	0.9277	0.9304	0.9270
Malignant vs. Benign	2	86%	89%	0.8270	0.8612	0.8032
MSLD	2	85%	79%	0.7913	0.8071	0.8006
ISIC 2019 and 2020 malignant or benign	2	89%	91%	0.9167	0.9232	0.9133

**Table 3:** Classification results using ResNet50 with individual dataset training.

Dataset Name	No. of Classes	Training Accuracy	Testing Accuracy	F1-Score	Sensitivity	Specificity
Skin Cancer ISIC	9	59%	55%	0.4422	0.4337	0.5701
ISIC 2019	9	61%	55%	0.3984	0.5585	0.3765
SKINL2	8	61%	67%	0.4017	0.4122	0.5615
HAM10000	7	76%	76%	0.4317	0.3837	0.5536
MSLD v2.0	6	74%	69%	0.6190	0.7101	0.6102
Skin Cancer Types Images	4	92%	100%	1.0000	1.0000	1.0000
PH2	3	44%	60%	0.6479	0.6944	0.7940
ISIC 2017	3	70%	60%	0.5442	0.5824	0.5297
Dermatology database used in MED-NODE	2	82%	66%	0.5846	0.5833	0.6071
Melanoma	2	93%	93%	0.9315	0.9312	0.9322
Malignant vs. Benign	2	89%	90%	0.8474	0.8219	0.8836
MSLD	2	82%	83%	0.8321	0.8461	0.8666
ISIC 2019 and 2020 malignant or benign	2	91%	92%	0.9210	0.9170	0.9293

**Table 4:** Classification results using MobileNetV3-small with individual dataset training.

Dataset Name	No. of Classes	Training Accuracy	Testing Accuracy	F1-Score	Sensitivity	Specificity
Skin Cancer ISIC	9	61%	58%	0.4040	0.5573	0.3853
ISIC 2019	9	57%	54%	0.4084	0.4118	0.4469
SKINL2	8	66%	60%	0.4541	0.5394	0.5469
HAM10000	7	75%	76%	0.4420	0.4012	0.5323
MSLD v2.0	6	71%	60%	0.5558	0.6560	0.5436
Skin Cancer Types Images	4	88%	97%	0.9653	0.9583	0.9772
PH2	3	67%	55%	0.6087	0.6277	0.6926
ISIC 2017	3	70%	67%	0.6293	0.6844	0.6100
Dermatology database used in MED-NODE	2	74%	44%	0.4375	0.4583	0.4625
Melanoma	2	91%	92%	0.9221	0.9217	0.9234
Malignant vs. Benign	2	88%	89%	0.8351	0.8072	0.8772
MSLD	2	79%	95%	0.9582	0.9615	0.9583
ISIC 2019 and 2020 malignant or benign	2	91%	92%	0.9213	0.9184	0.9264

**Table 5:** Classification results using DenseNet-161 with individual dataset training.

Dataset Name	No. of Classes	Training Accuracy	Testing Accuracy	F1-Score	Sensitivity	Specificity
Skin Cancer ISIC	9	64%	59%	0.5000	0.4814	0.6026
ISIC 2019	9	67%	59%	0.4103	0.5777	0.3944
SKINL2	8	72%	67%	0.5594	0.6564	0.5989
HAM10000	7	79%	78%	0.5001	0.4586	0.6011
MSLD v2.0	6	81%	80%	0.7253	0.7696	0.7052
Skin Cancer Types Images	4	92%	100%	1.0000	1.0000	1.0000
PH2	3	74%	60%	0.5750	0.6111	0.7348
ISIC 2017	3	72%	65%	0.6098	0.6626	0.5915
Dermatology database used in MED-NODE	2	87%	88%	0.8831	0.9166	0.875
Melanoma	2	94%	94%	0.9464	0.9457	0.9492
Malignant vs. Benign	2	91%	89%	0.8465	0.8391	0.8546
MSLD	2	86%	79%	0.7913	0.8006	0.8071
ISIC 2019 and 2020 malignant or benign	2	92%	92%	0.9281	0.9259	0.9315

**Table 6:** Classification results of all models with merged dataset training, across 12 consequent merging dataset cases.

Model Name	No. of Merged Datasets (Hyperdataset Name)	No. of Classes	Training Accuracy	Testing Accuracy	F1-Score	Sensitivity	Specificity
VGG16	2 (Merged dataset 1)	7	62%	50%	0.4865	0.4983	0.5652
ResNet50			62%	52%	0.5042	0.5436	0.5012
MobileNetV3-small			58%	40%	0.3721	0.4640	0.3616
DenseNet-161			67%	48%	0.4639	0.5144	0.4593
VGG16	3 (Merged dataset 2)	9	63%	48%	0.4807	0.5779	0.4660
ResNet50			63%	55%	0.5407	0.6589	0.5111
MobileNetV3-small			61%	56%	0.5349	0.6500	0.5296
DenseNet-161			67%	56%	0.5423	0.6444	0.5213
VGG16	4 (Merged dataset 3)	11	60%	52%	0.4628	0.4996	0.4785
ResNet50			60%	51%	0.4480	0.5015	0.4648
MobileNetV3-small			58%	50%	0.4872	0.5127	0.4877
DenseNet-161			65%	52%	0.4599	0.4974	0.4736
VGG16	5 (Merged dataset 4)	15	66%	52%	0.5581	0.5953	0.5608
ResNet50			66%	57%	0.5581	0.5868	0.6020
MobileNetV3-small			64%	49%	0.4383	0.4997	0.483
DenseNet-161			69%	55%	0.5941	0.6193	0.6339
VGG16	6 (Merged dataset 5)	20	69%	52%	0.5085	0.5891	0.5027
ResNet50			72%	60%	0.5881	0.6925	0.5773
MobileNetV3-small			66%	52%	0.5415	0.6355	0.5153
DenseNet-161			75%	60%	0.6120	0.6906	0.5942
VGG16	7 (Merged dataset 6)	21	72%	50%	0.5085	0.5891	0.5027
ResNet50			71%	60%	0.5881	0.6925	0.5773
MobileNetV3-small			68%	49%	0.5415	0.6355	0.5153
DenseNet-161			76%	55%	0.6120	0.6906	0.5942
VGG16	8 (Merged dataset 7)	26	63%	46%	0.4981	0.4876	0.6000
ResNet50			67%	52%	0.4424	0.4363	0.5185
MobileNetV3-small			64%	48%	0.4614	0.4495	0.5570
DenseNet-161			71%	53%	0.5356	0.5395	0.5728
VGG16	9 (Merged dataset 8)	27	51%	40%	0.4819	0.4728	0.5334
ResNet50			59%	49%	0.4394	0.4224	0.4915
MobileNetV3-small			56%	46%	0.3940	0.3860	0.4383
DenseNet-161			60%	51%	0.4609	0.4544	0.5243
VGG16	10 (Merged dataset 9)	28	63%	39%	0.4819	0.4928	0.5334
ResNet50			68%	46%	0.4394	0.4325	0.4915
MobileNetV3-small			66%	43%	0.4356	0.4660	0.4453
DenseNet-161			74%	49%	0.5009	0.5245	0.5409
VGG16	11 (Merged dataset 10)	28	68%	50%	0.4057	5.932	0.3651
ResNet50			74%	54%	0.4535	0.6472	0.3989
MobileNetV3-small			72%	52%	0.4040	0.5944	0.3497
DenseNet-161			78%	58%	0.5197	0.6762	0.4642
VGG16	12 (Merged dataset 11)	31	61%	30%	0.3247	0.4851	0.3031
ResNet50			68%	34%	0.3792	0.5412	0.3414
MobileNetV3-small			66%	33%	0.3352	0.5153	0.2931
DenseNet-161			73%	36%	0.4418	0.5525	0.4177
VGG16	13 (Merged dataset 12)	32	60%	31%	0.3170	0.5144	0.2830
ResNet50			67%	34%	0.3412	0.5266	0.2984
MobileNetV3-small			66%	33%	0.3274	0.5288	0.2774
DenseNet-161			72%	37%	0.4142	0.5486	0.3790

**Table 7:** Classification results of all models trained from scratch and with transfer learning, on the Skin Cancer ISIC dataset.

Model Name	Training Method	No. of Classes	Training Accuracy	Testing Accuracy	F1-Score	Sensitivity	Specificity
VGG16	From scratch	9	29%	24%	0.2168	0.3362	0.3120
ResNet50			40%	40%	0.3781	0.4373	0.4216
MobileNetV3-small			23%	10%	0.0834	0.2352	0.0665
DenseNet-161			50%	42%	0.3952	0.4549	0.4134
VGG16	Transfer learning	9	50%	43%	0.4247	0.4972	0.4482
ResNet50			61%	49%	0.4637	0.5338	0.4731
MobileNetV3-small			57%	41%	0.4030	0.5024	0.4250
DenseNet-161			66%	50%	0.4726	0.5095	0.4832

**Table 8:** Classification results of all models trained from scratch and with transfer learning, on the enhanced Skin Cancer ISIC dataset by using the Data Merger App.

Model Name	Training Method	No. of Classes	Training Accuracy	Testing Accuracy	F1-Score	Sensitivity	Specificity
VGG16	From scratch	9	66%	50%	0.3823	0.5800	0.3535
ResNet50			63%	50%	0.3764	0.5563	0.3539
MobileNetV3-small			64%	48%	0.3420	0.5036	0.3375
DenseNet-161			69%	55%	0.4162	0.6043	0.3848
VGG16	Transfer learning	9	58%	53%	0.3837	0.5670	0.3604
ResNet50			69%	58%	0.4256	0.5855	0.3969
MobileNetV3-small			68%	56%	0.4149	0.6125	0.3848
DenseNet-161			75%	61%	0.4537	0.6576	0.4106

**Table 9:** Sample per class for the original and the enhanced Skin Cancer ISIC dataset.

Dataset	Actinic Keratosis	Basal Cell Carcinoma	Dermatofibroma	Melanoma	Nevus	Pigmented Benign Keratosis	Seborrheic Keratosis	Squamous Cell Carcinoma	Vascular Lesion
Skin Cancer ISIC	114	376	95	438	357	462	77	181	139
Enhanced Skin Cancer ISIC	981	3739	334	11,910	1926	462	110	809	392

**Table 10:** Classification results of the ViT model trained with transfer learning, on the Skin Cancer ISIC dataset and the enhanced Skin Cancer ISIC dataset by using the Data Merger App.

Model Name	Dataset	Training Method	No. of Classes	Training Loss	Testing Accuracy	F1-Score	Sensitivity	Specificity
ViT	Skin Cancer ISIC	Transfer learning	9	0.4892	78.12%	0.6737	0.6899	0.6787
	Enhanced Skin Cancer ISIC			0.2294	91.87%	0.8037	0.7860	0.8394

**Table 11:** Classification results of ViT model and best-performing CNN (DenseNet-161) of test 2 on 32 classes of the merged dataset formed by using the Data Merger App.

Model Name	Training Method	No. of Classes	Training Loss	Testing Accuracy	F1-Score	Sensitivity	Specificity
ViT	Transfer learning	32	0.784	58%	0.5786	0.5908	0.6132
DenseNet-161			0.903	37%	0.4142	0.5486	0.3790

## Data Availability

The data presented in this study are available in public repositories referenced in Table 1. These data were derived from their original resources available in the public domain. The Data Merger App presented in the study is openly available in the GitHub repository at [https://github.com/MachineLearningVisionRG/DataMergerApp, accessed on 31 January 2025].
